# Supplementary material for: Unconventional short-range structural fluctuations in cuprate superconductors
Source: Sci Rep. 2022 Nov 28;12:20483. doi: 10.1038/s41598-022-22150-y (PMC9705399; doi:10.1038/s41598-022-22150-y)
Supplement: Supplementary file 1 — Supplementary Information. [file 41598_2022_22150_MOESM1_ESM.pdf]

Supplementary Information for

Unconventional short-range structural fluctuations in cuprate superconductors

D. Pelc, R. J. Spieker, Z. W. Anderson, M. J. Krogstad, N. Biniskos, N. G. Bielinski, B. Yu, T. Sasagawa, L. Chauviere, P. Dosanjh, R. Liang, D. A. Bonn, A. Damascelli, S. Chi, Y. Liu, R. Osborn, and M. Greven

Incommensurability of LTO peaks. The results presented in the main text show an emergent simplicity, yet there are a number of subtle points to be made, especially in comparison with previous work. Orthorhombic fluctuations in La-based cuprates have been studied fairly extensively *via* neutron scattering[1-4], which revealed two incommensurate peaks at doping levels above about 5% Sr and a commensurate response at lower doping. The incommensurability was related to the appearance of charge and spin stripes at lower temperatures[4], which are also incommensurate with the lattice. However, if the splitting of the diffuse peaks is taken to originate from a modulation of the LTO distortion, the spatial period of the modulation turns out to be larger than the LTO correlation length at high temperatures. In our simulations, we tested various real-space LTO tilt-angle configurations – a Gaussian dependence with either a sine or a cosine modulation, and the simple antiphase boundary discussed in the main text. With realistic correlation lengths and modulation periods, the only feature that is important for reproducing the observed incommensurability is the antiphase boundary: there is little difference between a simple step function (Eq. (1) in Methods) and a sine modulation, but a cosine modulation results in a commensurate peak. This implies that the neutron scattering experiments cannot distinguish between a true modulation and an antiphase boundary, and we therefore take the simplest possibility as the most likely. An appealing feature of the antiphase boundary scenario is that the incommensurability is solely determined by the LTO correlation length, *i.e.*, there is no need for an additional modulation length parameter. The commensurate LTO peaks previously observed in samples with low doping levels are then likely the result of a complex energy balance, where the energy gain from the antiphase boundary is no longer sufficient to lead to its formation (at least at the temperatures that have been accessed experimentally).

The role of atomic form factors. Whereas the neutron atomic form factors relevant to nuclear scattering do not depend on the scattering vector, the dependence is significant for X-rays. In principle, this can change the shape of the diffuse peaks and induce apparent peak shifts. However, the form factors[5] of Cu, O and La vary by at most 10-20% within the reciprocal space regions relevant for the LTO scattering, and therefore do not significantly affect the peak shapes and positions. We have checked this by explicitly including the wavelength dependence of the form factors in the simulation and found no obvious shifts of the diffuse peaks.

Anisotropy and shifts of the LTO superstructure peaks. The LTO peaks are markedly anisotropic in reciprocal space, especially for underdoped compounds (Supplementary Fig. S1). This is not surprising, since the LTO tilt has a well-defined direction, and there is no *a priori* reason for the correlation lengths parallel and perpendicular to the tilt direction to be the same. We find that the anisotropy does not significantly depend on temperature, and that it is highest for the sample with 8% Sr, where the ratio of short and long diffuse peak widths is about 1.8.

In X-ray data, we noticed a slight shift of the LTO diffuse peaks away from the nominal (H+1/2 K+1/2 L) positions (in tetragonal notation). Notably, the superstructure Bragg peaks below T_{LTO} are not shifted within the experimental resolution. The shifts are partially the result of an imperfect refinement of the X-ray data, *i.e.*, inaccuracies in the sample orientation matrix. Yet these also induce shifts of the regular Bragg peaks, and the LTO peaks remain slightly shifted even when this is corrected for (Supplementary Fig. S2). As noted above, the X-ray atomic form factors do not cause appreciable shifts. An intrinsic reason for the shifts could be a small local expansion of the lattice in the LTO-distorted region; this is not taken into account in our simulation, which assumes that the volume of the orthorhombic unit cells is always the same, independent of tilt angle. Of course, this assumption is not completely realistic: the long-range-ordered LTO phase has distinct a and b lattice parameters, and orthorhombicity should appear in the short-range distorted regions, leading to local lattice parameters that are slightly different from the tetragonal matrix. It is, however, not trivial to include such an effect in our simple simulation, and it would cause an elastic coupling between the orthorhombic regions and the tetragonal surroundings, as well as an effective coupling between different ordered regions. The shifts should be reproducible with a more sophisticated calculation that takes the local unit-cell size into account.

We note also that the shifts may support the rare-region picture; namely, if the LTO distortion were approximately homogeneous in real space, it would have to exhibit the same lattice constants as the tetragonal unit cell (or, equivalently, the entire lattice would need to expand), and the peaks would always appear at the nominal positions. In contrast, if the regions with LTO tilts are far apart, a local lattice expansion can be easily accommodated.

Estimate of distance between distorted regions. A rough estimate of the spatial distance between distorted regions can be obtained by comparing the intensity of the low-temperature LTO superstructure peak to the diffuse intensity above the LTO transition. We simulate the intensities using the same approach as for the diffuse scattering calculation in Fig. 2. We assume that kinetic diffraction theory is still valid for the sharp superstructure peaks. The calculation therefore likely overestimates the Bragg peak intensity, and thus provides a lower limit to the rare-region distance. From the experiment, the ratio of the low-temperature (30 K) to the high-temperature (approximately 500 K) integrated intensities is about two orders of magnitude, as seen most clearly for the sample with $x = 0.12$ in Fig. 3. To estimate the distance, we calculate the intensity ratio for long-range (constant tilt angle) and short-range (Gaussian tilt angle profile) LTO distortions on numerical grids of different sizes and find the grid size for which the ratio matches with

experiment. The linear size of the grid is then a measure of the average distance among distorted regions for periodic boundary conditions. Assuming that the amplitude α_0 of the Gaussian tilt angle profile is the same as the long-range constant tilt, agreement with the experimental intensity ratio is obtained for a linear grid size of about 60 orthorhombic unit cells for a short-range LTO correlation length of four unit cells.

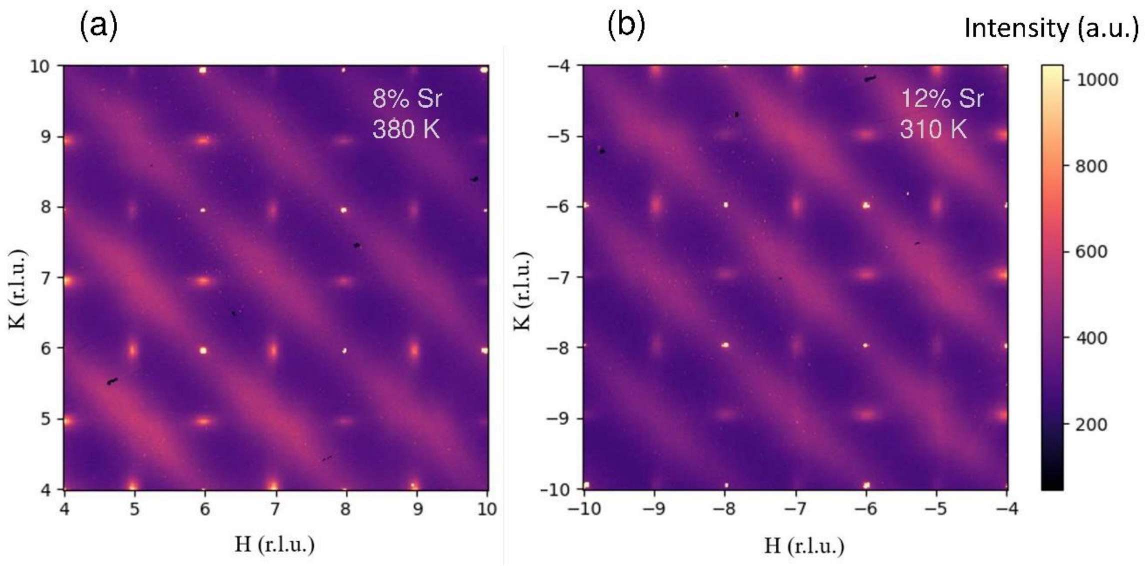

Supplementary Fig. S1: Anisotropy of LTO diffuse scattering. X-ray diffuse scattering for LSCO with (**a**) x = 0.08 and (**b**) x = 0.12, at 380 K and 310 K, respectively, showing a pronounced anisotropy in the *HK* plane. The data are shown in the orthorhombic reciprocal lattice, so that the diffuse peaks are at integer Bragg positions, in contrast to Fig. 1, where the high-temperature tetragonal lattice is used.

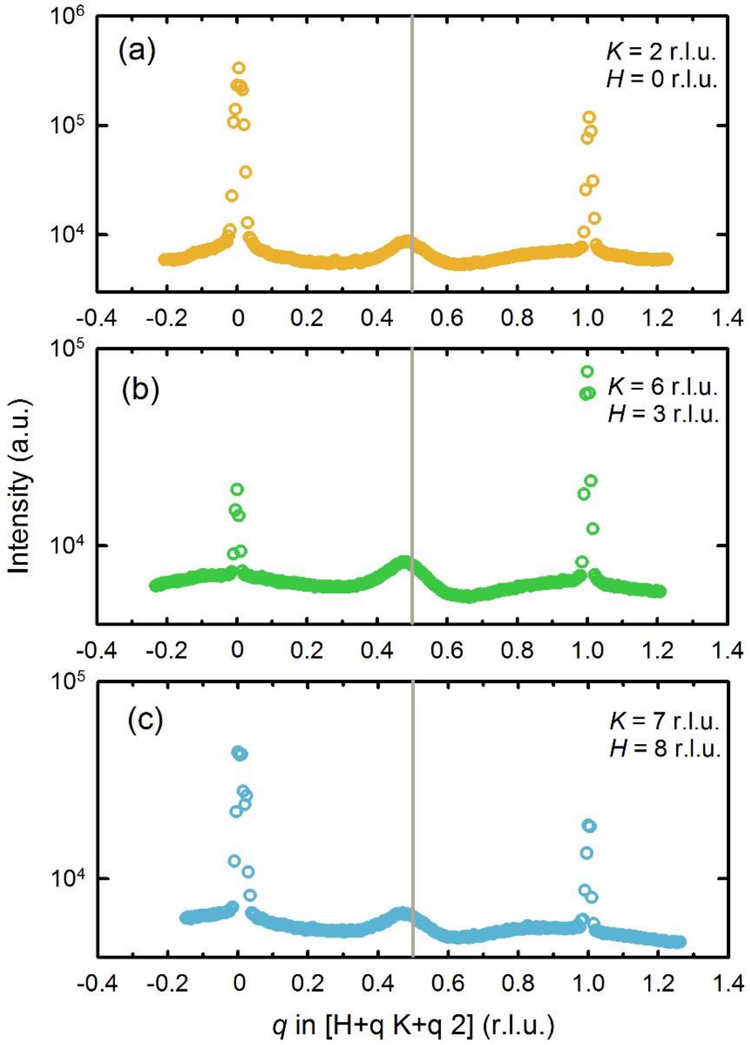

Supplementary Fig. S2: Shift of the LTO diffuse peak. X-ray diffuse scattering for a LSCO sample with $x = 0.2$ at 200 K, in three different Brillouin zones, with zone centers (**a**) (*H K L*) = (0 2 2); (**b**) (*H K L*) = (3 6 2); (**c**) (*H K L*) = (7 8 2). The diffuse peaks are slightly shifted from the nominal (*H*+1/2 *K*+1/2 *L*) positions, with the shifts more obvious in higher zones.

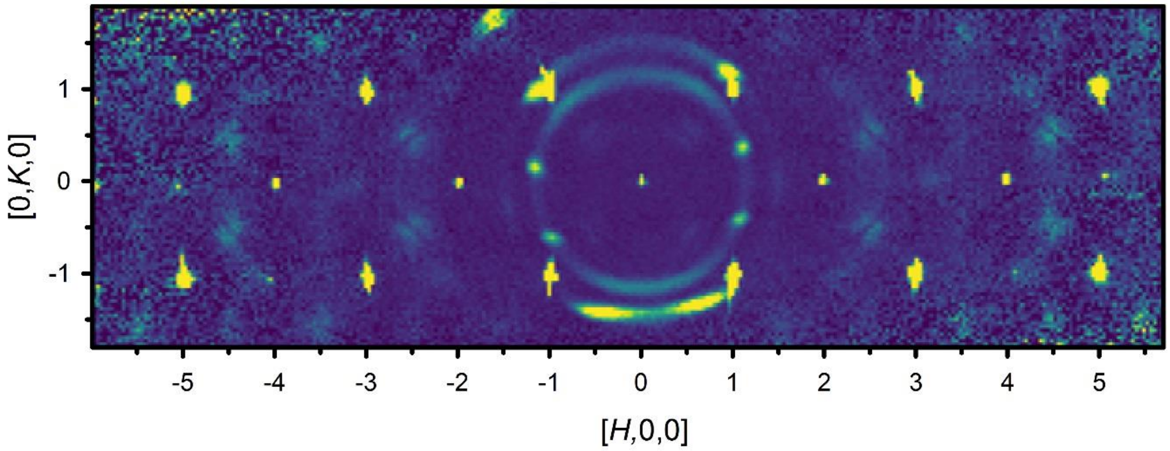

Supplementary Fig S3: Expanded view of the L = 4 r.l.u. plane of a LSCO sample with 20% Sr (data obtained with the CORELLI instrument). The split LTO diffuse peaks are clearly visible, and are clearest in this plane due to a combination of structure factor and counting statistics. The rings and additional peaks in the first and second zone are from the aluminum sample holder and sample environment.

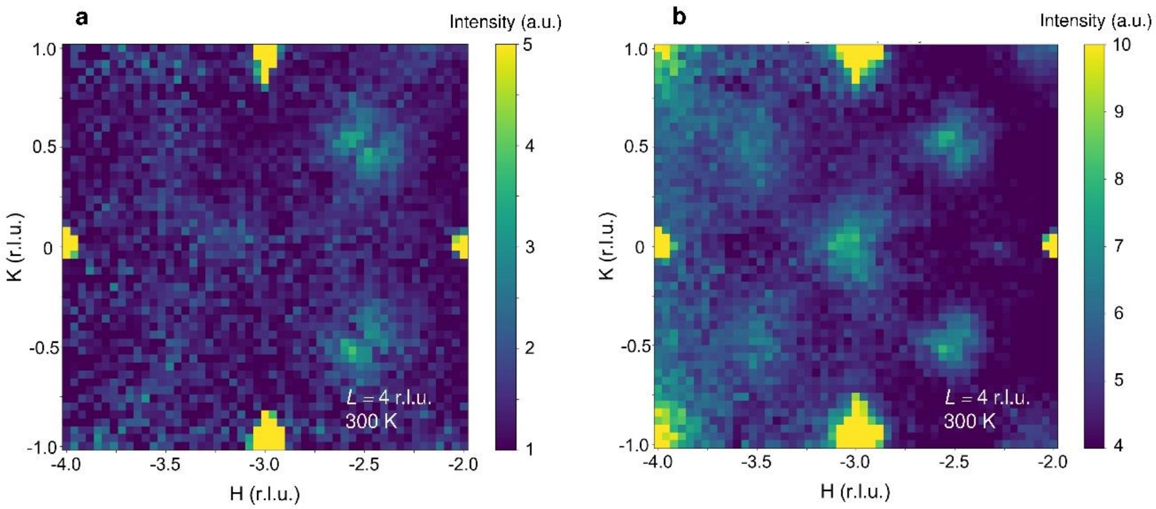

Supplementary Fig. S4: Comparison between quasielastic and total diffuse neutron scattering obtained with the CORELLI instrument. (**a**) diffuse scattering with cross-correlation (quasielastic, <0.5 meV energy transfer) for the LSCO sample with x = 0.2 (same as Fig. S3). The incommensurate LTO peaks are clearly visible, with a barely discernible signal around the (-3 0 4) forbidden Bragg position that corresponds to LTLO tilts. (**b**) total scattering (<10 meV energy transfer, with complex weighting) in the same reciprocal space region as in (a), with significantly stronger signal at the LTLO position.

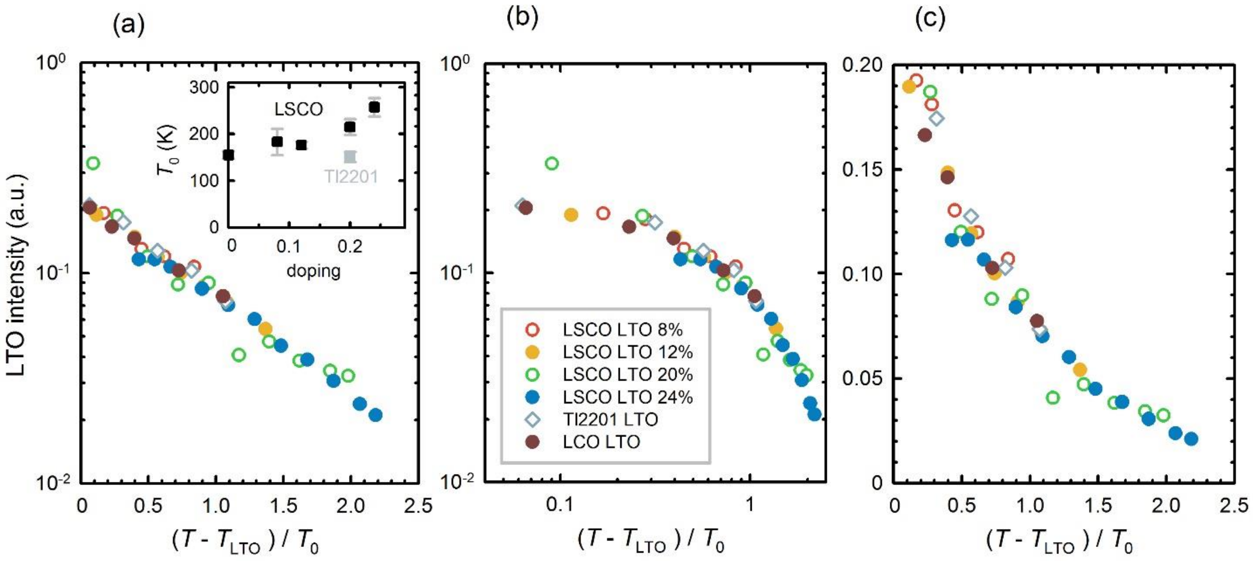

Supplementary Fig. S5: Scaling of the integrated LTO peak intensity. (**a**) Log-linear plot of the scaled intensities. In contrast to Fig. 4, the temperature scale is normalized by the best-fit exponential decay rates T_0 for each sample (for T_0 values see Table S1 and inset). A relatively small systematic increase of T_0 with doping is observed for LSCO. (**b,c**) The same data as in (a) on log-log and linear scales, respectively. The temperature dependence is clearly not consistent with a power law in any meaningful temperature range.

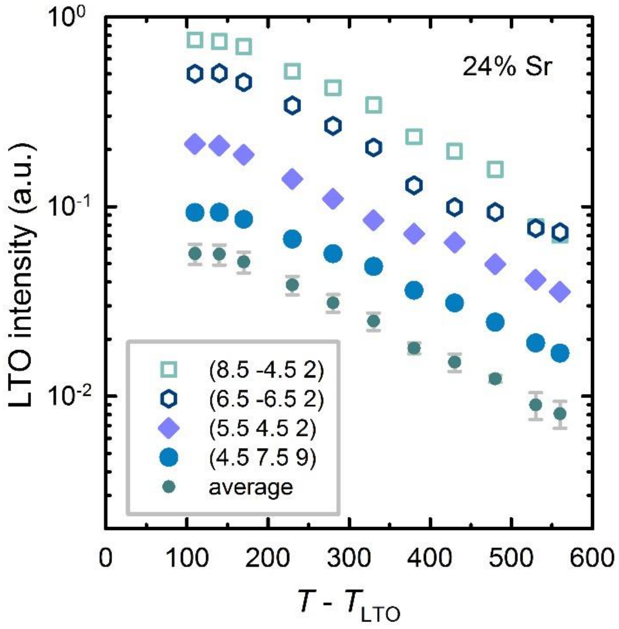

Supplementary Fig. S6: Temperature dependence of the LTO peak intensity in different Brillouin zones, for a sample with 24% Sr. The numbers in parentheses denote (*HKL*) in tetragonal reciprocal lattice units, and the average is shown with error bars that correspond to 1 s.d. Data have been shifted for clarity.

Compound	T_0 (K)
LCO	155 ± 8
LSCO 8%	183 ± 28
LSCO 12%	176 ± 7
LSCO 20%	215 ± 17
LSCO 24%	257 ± 20
Tl2201	151 ± 11

Supplementary Table 1 | Exponential decay rate T_0 for each sample. T_0 corresponds to the average best-fit exponential decay rate of three different temperature fitting ranges. The uncertainty was estimated to be the standard deviation from the mean (for LSCO 12%, the largest individual fit uncertainty was used since it was larger than the standard deviation).

Supplementary References

[1] Kimura, H., Hirota, K., Lee, C.-H., Yamada, K. & Shirane, G. Structural instability associated with the tilting of CuO_6 octahedra in $La_{2-x}Sr_xCuO_4$. *J. Phys. Soc. Jpn*. **69**, 851-857 (2000)

[2] Braden, M. et al. Analysis of the local structure by single-crystal neutron scattering in $La_{1.85}Sr_{0.15}CuO_4$. *Phys. Rev. B* **63**, 140510(R) (2001)

[3] Wakimoto, S., Lee, S., Gehring, P. M., Birgeneau, R. J.& Shirane, G. Neutron scattering study of soft phonons and diffuse scattering in insulating $La_{1.95}Sr_{0.05}CuO_4$. *J. Phys. Soc. Jpn.* **73**, 3413-3417 (2004)

[4] Wakimoto, S. et al. Incommensurate lattice distortion in the high temperature tetragonal phase of $La_{2-x}(Sr,Ba)_xCuO_4$. *J. Phys. Soc. Jpn.* **75**, 074714 (2006)

[5] Brown, P. J., Fox, A. G., Maslen, E. N., O'Keefe, M. A. & Willis, B. T. M. *International Tables for Crystallography* Vol. C, ch. 6.1, pp. 554-595 (2006)
